# Nanoscale Wear and Mechanical Properties of Calcite: Effects of Stearic Acid Modification and Water Vapor

**DOI:** 10.1021/acs.langmuir.1c01390

**Published:** 2021-08-06

**Authors:** Natalia A. Wojas, Illia Dobryden, Viveca Wallqvist, Agne Swerin, Mikael Järn, Joachim Schoelkopf, Patrick A. C. Gane, Per M. Claesson

**Affiliations:** † Bioeconomy and Health Division, Department of Materials and Surface Design, RISE Research Institutes of Sweden, Box 5607, SE-114 86 Stockholm, Sweden; ‡ Division of Surface Chemistry and Corrosion Science, Department of Chemistry, School of Engineering Sciences in Chemistry, Biotechnology and Health, KTH Royal Institute of Technology, Drottning Kristinas väg 51, SE-100 44 Stockholm, Sweden; § Division of Materials Science, Department of Engineering Sciences and Mathematics, Luleå University of Technology, SE−971 87 Luleå, Sweden; ∥ Department of Engineering and Chemical Sciences: Chemical Engineering, Faculty of Health, Science and Technology, Karlstad University, SE-651 88 Karlstad, Sweden; ⊥ Omya International AG, Baslerstrasse 42, CH-4665 Oftringen, Switzerland; # Department of Bioproducts and Biosystems, School of Chemical Engineering, Aalto University, P.O. Box 16300, FI-00076 Aalto, Finland

## Abstract

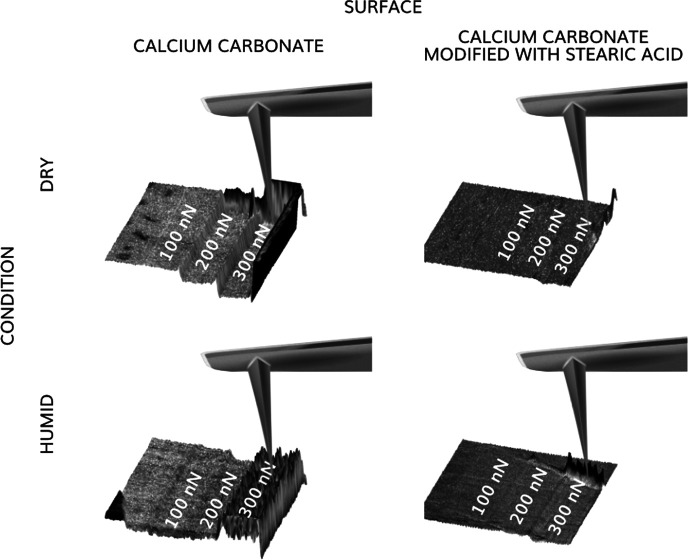

Understanding the wear of mineral fillers is crucial for controlling industrial processes, and in the present work, we examine the wear resistance and nanomechanical properties of bare calcite and stearic acid-modified calcite surfaces under dry and humid conditions at the nanoscale. Measurements under different loads allow us to probe the situation in the absence and presence of abrasive wear. The sliding motion is in general characterized by irregular stick-slip events that at higher loads lead to abrasion of the brittle calcite surface. Bare calcite is hydrophilic, and under humid conditions, a thin water layer is present on the surface. This water layer does not affect the friction force. However, it slightly decreases the wear depth and strongly influences the distribution of wear particles. In contrast, stearic acid-modified surfaces are hydrophobic. Nevertheless, humidity affects the wear characteristics by decreasing the binding strength of stearic acid at higher humidity. A complete monolayer coverage of calcite by stearic acid results in a significant reduction in wear but only a moderate reduction in friction forces at low humidity and no reduction at 75% relative humidity (RH). Thus, our data suggest that the wear reduction does not result from a lowering of the friction force but rather from an increased ductility of the surface region as offered by the stearic acid layer. An incomplete monolayer of stearic acid on the calcite surface provides no reduction in wear regardless of the RH investigated. Clearly, the wear properties of modified calcite surfaces depend crucially on the packing density of the surface modifier and also on the air humidity.

## Introduction

Calcium is the fifth most common element of the earth’s crust^1^ and is abundant in the form of calcium carbonate (CaCO_3_) in rocks, such as chalk, limestone, and marble.^1,2^ CaCO_3_ plays a part in a wide range of industrial applications. The most common crystalline form of CaCO_3_ used both in laboratory studies and in industry is calcite. Aragonite is less commonly used, and the third polymorph, vaterite, is unstable at normal temperature and pressure.^3−5^ There are two types of CaCO_3_ granulates widely used in industry, ground calcium carbonate, GCC,^5,6^ and precipitated calcium carbonate, PCC, which usually is obtained by carbonation in water of calcium hydroxide in the form of slaked burned lime.^5,7^ The filler market for this fine mineral is not only related to cost reduction, but increasingly in functional products, covering a variety of requirements in applications within packaging, plastics, pharmaceuticals, water treatment, and so on.^1,6^ However, depending on the final goods’ requirements, untreated CaCO_3_ cannot always be directly used, such as when mixed with low-surface-energy organic materials.^1^ The hydrophilic CaCO_3_ surface is not compatible with hydrophobic polymers; therefore, surface modification of calcite must be performed.^1^

To achieve good adhesion and effective mixing between filler and polymer matrix, their surface energies should be close to each other.^8^ The most frequently used coupling agents with calcium carbonate surface are based on fatty acids,^1,9−12^ but silanes,^13,14^ zirconates,^1,13,14^ and titanates^1,13,15^ can also be considered. Stearic acid is one of the most commonly used fatty acid surface modifiers on CaCO_3_ surfaces,^9−11^ acting to decrease the surface energy of uncoated CaCO_3_ mineral fillers from about 48–60 mJ/m^2^^16−18^ down to 21–32 mJ/m^2^, depending on the packing density of stearic acid,^16−20^ close to the value of many polymeric matrices.^8^ Such treatment influences not only the dispersibility but also mechanical properties (tensile strength, elongation and elastic modulus, stiffness, viscosity, abrasion resistance, thermal and flame retardance, etc.).^1,13,21,22^ However, industrial surface modification is not a straightforward operation. To optimize the balance between desired parameters and process costs, the amount of fatty acid should be controlled. A too low amount reduces the surface resistance and exposes highly undesirable hygroscopic CaCO_3_ areas,^23^ whereas an excessive amount of organic material can lead to inferior mechanical properties, processing problems, and high costs.^24,25^ In addition to the complexity of the treatment, one should also consider degradation of the coated surface. Surface-modified calcite as well as final coatings, and primarily polymer filler applications, are exposed to mechanical stresses and chemical changes leading to local wear, where the environment affects the severity of the wear process.^23,26^ In this work, we focus on the wear resistance of the modified calcite surface in humid and dry air. This is of particular relevance to processing with hydrophobic polymers, such as poly(vinyl chloride) (PVC) and polyolefins, where the system is highly sensitive to the quality of surface treatment and the presence of humidity.

The local wear resistance of adsorbed fatty acid layers on calcite, and how it is affected by the environment, has not been clarified. So far, in particular, the initiation of wear on a local scale on modified calcite surfaces has not been considered in detail, although the atomic force microscopy (AFM)-based method itself for assessing local wear has been used for other organic systems and is considered as well suited.^27,28^ To this end, we prepared and characterized unmodified and stearic acid surface-modified rhombohedral CaCO_3_ mineral surfaces using stearic acid vapor exposure. Both fully covered surfaces and surfaces coated with a patchy stearic acid layer were considered. The wear resistance and nanomechanical properties of calcite were measured and evaluated by the highly surface-sensitive atomic force microscopy technique. Our study offers a deeper understanding about local wear properties of unmodified and modified calcite surface under air exposure and in contact with water vapor. To our knowledge, no similar study has been reported before, but we note a recent study on calcite friction in aqueous solutions,^29^ where effects of different cations present in solution were highlighted. Surface forces between two rough calcite surfaces in aqueous solution have also been investigated by means of a surface force apparatus^30^ and AFM,^31^ and we have previously investigated the effect of humidity on adhesive and long-range forces between an AFM tip and calcite in air.^23^

## Materials and Methods

### Materials

The calcite material analyzed was optical-quality Iceland spar (purchased from Geocity AB, Stockholm, mined in Madagascar) cleaved to provide a uniform fresh surface with stainless steel chisel and hammer,^23^ following the natural crystal angles of the rhombohedron along the dominant {101̅4} cleavage plane. This highly hygroscopic and wettable surface^32,33^ was immediately purged with pressurized nitrogen (industrial quality: nitrogen ≥99.9 vol %, oxygen ≤20 ppm, water ≤10 ppm). At least three samples for each study were analyzed, each being a few millimeters thick with an approximate surface area of 50–100 mm^2^, and with no evidence of microcracks or excessive surface steps. Epoxy glue (Bostik, France) was used for sample attachment to the magnetic disk used in the AFM studies. A sapphire calibration sample (Bruker) was used for deflection sensitivity calibration of the AFM probes, and a titanium roughness sample (RS-12M, Bruker) for the determination of the probes’ tip end radii. Stearic acid (C_18_) (Sigma-Aldrich, ≥97.0%) was used for modification of the calcite surface.

### Surface Modification

The CaCO_3_ surface was modified by exposure to saturated stearic acid vapor. Such modification is possible only at a high enough vapor pressure, which strongly depends on temperature.^34^ For this reason, the modification has to be performed above the melting temperature (69.3 °C for stearic acid^35^). The humidity inside the oven (UF 55, Memmert) was estimated to be well below 2.5% relative humidity (RH), and at such low RH, the calcite recrystallization is retarded.^23^ The samples were kept in an unsealed 1.5 L glass box for one of the time periods: 10 min or 4 h at a temperature of 105 °C. The box contained a glass beaker filled with 20–25 g of stearic acid that was previously melted at the same temperature for about 90 min.

### Wear Resistance and Surface Characterization

The wear resistance, topography, and nanomechanical properties of freshly cleaved, aged, and coated calcite surfaces were recorded by utilizing a Nanoscope V Controller connected to a Multimode 8 AFM with a standard scanner (S/N: 10578JVLR, Bruker) using hard diamond-like carbon-coated HQ:NSC35/Hard/Al BS probes (Mikromasch) with a nominal tip diameter of <20 nm, a resonance frequency of about 300 kHz, and a spring constant of 16 N/m. The normal spring constant, *k*_z_, calculated using the thermal tune method within the Nanoscope program was determined to be 18.6 ± 1.7 N/m. The torsional spring constant, *k*_t_, was calculated to be (8.5 ± 0.7) × 10^–8^ N m/rad following the method proposed by Álvarez-Asencio et al.^36^ The deflection sensitivity in the normal direction was found to be 25.1 ± 1.1 nm/V with use of the sapphire calibration sample, while by utilizing a polycrystalline titanium roughness sample, the tip radius of the AFM probe was determined to be 6.1 ± 0.4 nm at a 0.5 nm indentation depth and 17.2 ± 2.3 nm at a 5 nm indentation. The tip radius was found to change slightly during contact mode wear measurements with the calcite surface when applying high forces using long sliding distances. To minimize this effect to below a limit of 20% of the nominal radius at 0.5 nm indentation depth (and 6% at 5 nm), a new tip was used for each sample.

The samples were studied under controlled relative humidity (RH) set at <5 and 75 ± 3%RH, using a gas flow rate of 0.8 ± 0.1 L/min. The desired RH was achieved by connecting pressurized nitrogen (overpressure of 13.8 ± 3.5 kPa) to a P-50 membrane flow humidifier (Cellkraft AB). The outlet stream was fitted with a heated tube near the AFM probe holder, although without direct contact, allowing the stream to easily leave the holder. Ultrapure Milli-Q water (type 1, ASTM D 1193-91) was used for creating the humid medium. The RH of the gas entering the AFM environment was measured with an external sensor (HMT317, Vaisala), placed near the calcite samples. The laboratory room temperature was 23 ± 0.5 °C, and the room humidity was 24–30%RH.

The modified calcite surfaces were studied immediately after preparation. Each experiment was carried out in a closed cell under constant nitrogen purging at controlled humidity following the cycle: dry nitrogen (<5%RH), followed by 30 min exposure to humid nitrogen vapor (∼75%RH), and next, by up to 60 min of dry nitrogen exposure (<5%RH), where at each step, the same nominal deflection setpoint forces were applied: 100, 200, and 300 nN. This allowed us to see the impact of the humidity condition, the reversibility of the effect, and to consider possible effects of changes in the tip radius of the probe. The deflection setpoint [V], *D*_SP_, can be calculated from
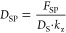
1where *F*_SP_ is the force setpoint [nN]; *k*_z_ is the normal spring constant [N/m]; and *D*_S_ is the deflection sensitivity [nm/V]. To achieve the right force setpoint (expected error of maximum 0.02 V), it is crucial to set the deflection signal of the photodetector to exactly 0.00 V prior to measurements.

Nanomechanical properties were determined using 512 × 512 pixel images over a scanned area of 2 × 2 μm^2^ recorded in PeakForce Quantitative Nano-scale Mechanical (PF QNM) mode. This allows simultaneous capturing of images showing height, tip–sample adhesion, and sample deformation. For wear measurements in contact mode, the scan size was lowered to 1 × 0.67 μm^2^ (512 × 340 pixels) to minimize the damage of the tip. The load applied to the calcite surface during wear measurements was increased stepwise from 100, via 200, and through to 300 nN. The worn region is located in the central area of the subsequent topographical and nanomechanical images. The scanning frequency was kept constant at 1.0 Hz, and during imaging, the scanning angle was at times changed from standard 90–0° to check for possible image artifacts.

The friction data were further analyzed using the NanoScope Analysis program and ForceIT software in Matlab. In all cases, no image enhancement was performed apart from first-order plane fitting and flattening (with marked excluded area of wear) of the height channel. The friction force was extracted using the following procedure. First, half of the difference in lateral photodetector signal, Δ*V*/2, between the two scanning directions for each scan line (1 μm) was calculated. Here, 50 nm at each end was removed to exclude effects resulting from the change in the scan direction. Next, the average value of Δ*V*/2 was obtained from all lines at a given applied force as calculated using eq 2, where the friction force, *F*_f_, was quantified^37^
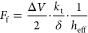
2where δ (3420 V/rad) is the lateral photodetector sensitivity and *h*_eff_ is the effective height of the probe (here determined to be 15 μm).

The coefficient of friction, μ, was calculated from the slope of the curve showing friction force as a function of load.^37^

## Results and Discussion

### Unmodified Calcite

**Figure 1:**
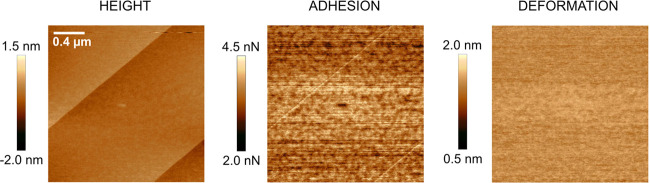
Topography, adhesion, and deformation images of a freshly cleaved calcite surface recorded before wear measurements obtained at a relative humidity below 5% at imaging load of 20 nN. The data were collected using PeakForce QNM mode. The image size is 2 × 2 μm^2^ and contains 256 × 256 data points.

**Figure 2:**
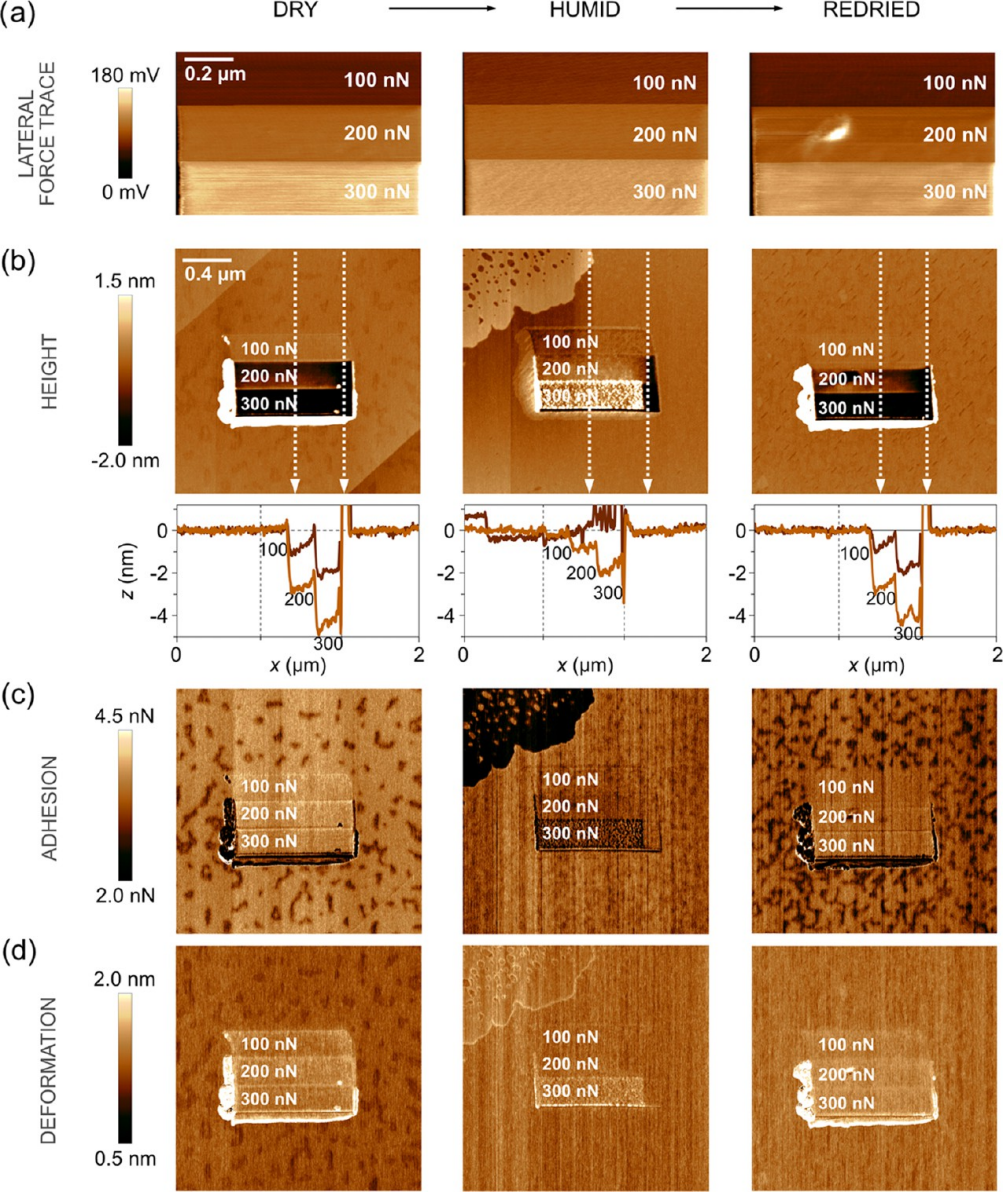
(a) Lateral force images, expressed in terms of lateral photodetector voltage, recorded during the wear measurements of freshly cleaved calcite at dry, humid, and redried condition and increasing applied force. The sliding speed was 1 μm/s. The scan size is 1 × 0.67 μm^2^. (b) Topography images, followed by line scans across the worn area that show the wear depth; below (c) adhesion images, and at the bottom row, (d) deformation images determined after the wear measurements. The left column shows data obtained at below 5%RH, the middle column data obtained after increasing to 75%RH, and the right column data obtained after again drying to <5%RH. The scan size is 2 × 2 μm^2^, and the images contain 512 × 512 data points. Average roughness, adhesion, and deformation of worn and unworn areas can be found in Tables S1–S3 in the Supporting Information.

PeakForce QNM images, illustrating topography, adhesion, and deformation of freshly cleaved calcite (water contact angle below 10°), which were recorded before wear measurements, are shown in Figure 1. The topographical image shows typical surface features including clear steps on the surface, also small trenches about 0.3 nm deep in the flat regions. The trenches, more clearly seen in Figure 2, are most likely associated with high stress on the most outer surface caused by the cleaving procedure,^38^ and clear images of these have been reported and discussed in detail in our previous publications.^23^

A freshly cleaved calcite surface was challenged by the AFM tip under the combined action of load and shear. As the tip traverses the calcite surface, the cantilever laterally bends due to friction forces. The resulting lateral force was monitored by measuring the lateral photodetector response, and the data are shown in the top row of Figure 2a under effective dry condition (<5%RH) and under humid condition (75%RH), and again after returning to below 5%RH. The effect on the calcite surface was monitored in PeakForce QNM mode, and Figure 2b–d displays the sample topography, tip–sample adhesion, and sample surface deformation determined after the wear measurements.

**Figure 3:**
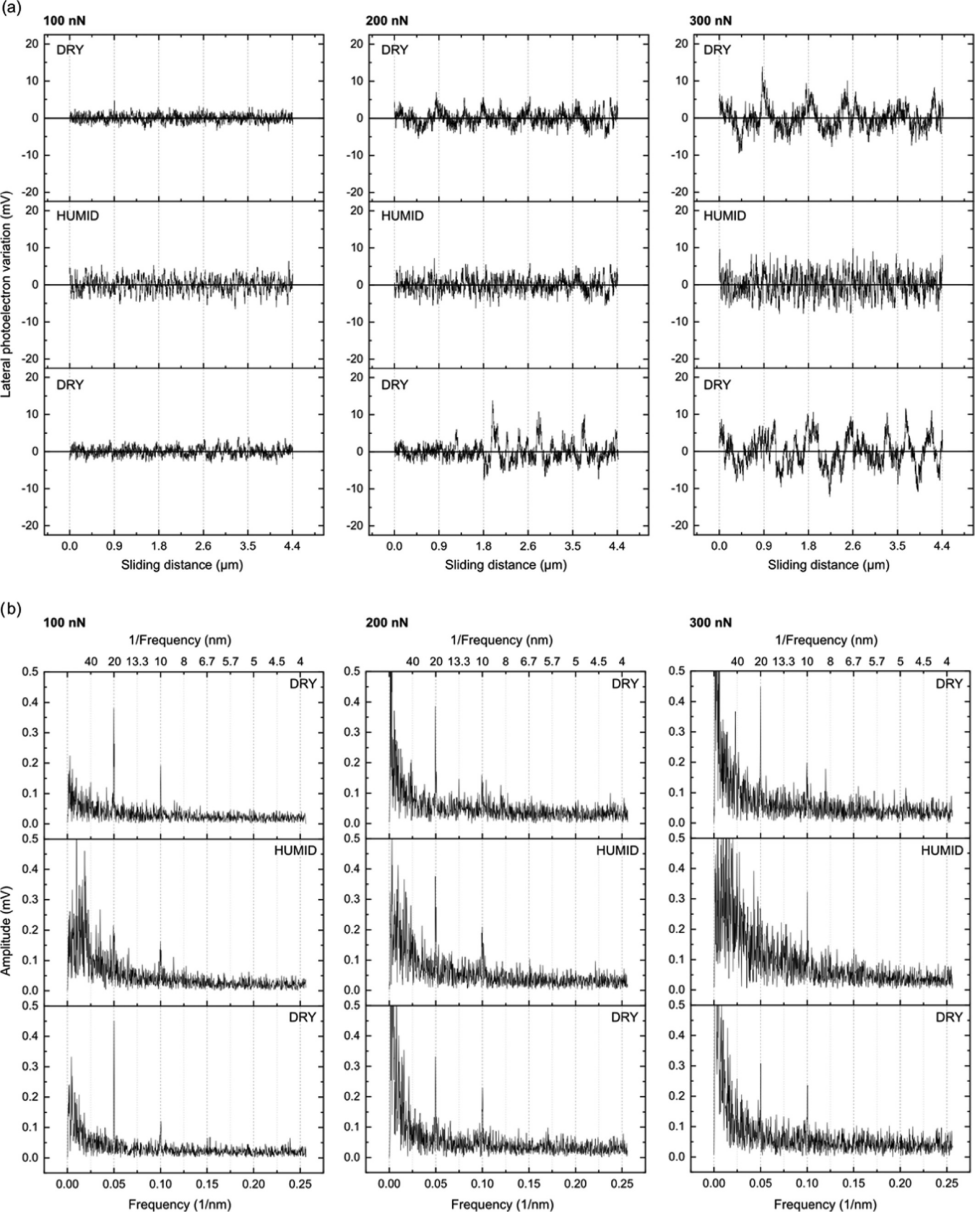
(a) Lateral force variations and (b) one-dimensional Fourier transforms of the data reported for freshly cleaved calcite under <5%RH (dry), 75%RH (humid), and again <5%RH (redried) conditions. The first column contains data recorded at a load of 100 nN, middle column data at 200 nN, and third column data at 300 nN. The sliding speed was 1 μm/s in all cases.

The worn area can clearly be distinguished in the middle of these images showing typical surface features on calcite. At a load of 100 nN, the wear is very small, but we note that the trenches, seen as dark regions in topography, adhesion, and deformation images outside the worn area, have disappeared. As the load is first increased to 200 nN and then to 300 nN, clear wear scars due to abrasive wear are observed, and the worn material has to a large extent accumulated by deposition at the edges of the worn area. The loose wear material extends from the surface and displays larger deformation and lower adhesion to the tip compared to unworn calcite. In the lateral force images (Figure 2a), one can clearly distinguish the surface areas that have been exposed to the different loads, which is due to the load dependence of the friction force. However, one can also see some variations over the areas that have been exposed to the same load, and this will be discussed further in relation to Figure 3.

Below 5%RH, we notice that significant abrasive wear occurs at loads of 200 and 300 nN, and the material released is pushed to the side of the worn area. The worn area appears relatively homogeneous even though the wear depths at the highest load approaches 4 nm. The situation is different at 75%RH. Some worn material is still pushed to the side, but clearly less so than in the dry state, and now many small wear particles are found inside the worn area. Further, the wear depth is smaller at 75%RH than in the dry state. When the sample is dried again to below 5%RH, a similar wear pattern is observed as in the initial dry state. Thus, we reach the conclusion that adsorbed water changes the wear characteristics of the calcite surface.

One could expect that adsorbed water reduces the friction force, but as we will show below, the friction force at 75%RH is only marginally lower than that obtained at below 5%RH, so there must be another reason for the different wear characteristics found at the two humidities. It is known that water adsorbs increasingly to hydrophilic surfaces such as calcite when the relative humidity is increased. Different studies of water adsorption on calcite suggest either a patchy water adsorption layer^39,40^ or a continuous layer,^41,42^ where results from our previous study were consistent with the idea of a continuous water layer.^23^ The water layer thickness under humid conditions has been reported to reach about 1.5 nm.^39−42^ Thus, at 75%RH, we expect a water layer thickness of about 1 nm. This amount of water is not very large, but sufficient for allowing capillary forces to develop between the hydrophilic wear particles and between the wear particles and the hydrophilic calcite surface, as well as between the tip and the surface. The presence of capillary forces between wear particles and between wear particles and the calcite surface is the reason why at 75%RH the wear particles are more difficult to push to the side, and instead have a higher tendency to remain in the worn area. Since the wear particles tend to remain in the worn area, they effectively reduce the average wear depth at higher humidity. Nonetheless, the worn area containing the wear particles remains locally significantly rougher than the virgin calcite surface. Based on the rapid reaction of the calcite surface with water at high humidity, it seems plausible that the wear particles to a large extent are built up from the hydrated form of calcite.

In our previous work,^23^ we demonstrated that the recrystallized form of calcite can be distinguished from pristine calcite by a lower adhesion force. In the adhesion images in Figure 2c, we can clearly distinguish such areas at 75%RH in the top left corner, where a layer of hydrated calcite is present. We note that exposure to 75%RH has resulted in a significant increase in the area fraction that is covered by recrystallized calcite, which is due to the dissolution and recrystallization process that occurs more rapidly at higher humidities. This is most clearly seen by comparing the adhesion images at 5%RH before and after exposure to 75%RH, where the fraction of the surface covered by low adhesion areas (recrystallized calcite) has increased after exposure to high humidity. We note that at 5%RH, no recrystallized calcite remains in the worn area, i.e., the recrystallized regions are easily worn off. In the deformation images of Figure 2d, we note the higher deformation of the worn material than that of the bare calcite, which is a consequence of the wear particles not being strongly attached to each other.

We now take a closer look at the friction traces, and in Figure 3, we have combined five of them for each condition. By inspecting the data obtained at a load of 100 nN, where abrasive wear is insignificant, we note a higher amplitude of the variation in the lateral force at 75%RH. We will refer to this as a higher stick-slip amplitude (i.e., the variation between the depth of a valley and the height of a peak). This observation suggests that in the presence of water, the calcite surface becomes slightly softer, which leads to a higher propensity for the tip to stick to the surface. Alternatively, one could interpret the large stick-slip amplitude to be due to capillary condensation between the tip and the surface. However, one would expect this to also lead to a larger friction force as the capillary is dragged across the surface, but this is not consistent with our data that show similar friction force at <5 and 75%RH at a load of 100 nN. To evaluate if there is a characteristic stick-slip length, we carried out a one-dimensional Fourier transform, FT, analysis, and the results are shown in the lower part of Figure 3. We observe a major sharp peak at 0.05 nm^–1^, which corresponds to a sliding distance, *d*, of 20 nm and an oscillatory frequency of 50 Hz, i.e., the same as the AC frequency in the electrical power lines. Thus, we assign this peak and its overtones to electrical noise that carries no information. No other clear peaks are observed, which suggests that unlike for polymer surfaces, calcite does not undergo plastic deformations that give rise to characteristic stick-slip lengths.^43−47^ However, we note that in the humid state, the FT amplitude increases more at low values of 1/*d* compared to that in the dry state. Thus, in the humid state, there is a greater probability for a larger stick-slip length, which is consistent with the larger stick-slip amplitude.

At higher forces, we observe a few large peaks that appear in the lateral force trace. We suggest that these large stick-slip events give rise to initiation of abrasive wear. They are more pronounced at 5%RH than at 75%RH, which suggests that the presence of water reduces the brittleness of the calcite surface and whereby counteracts abrasive wear. This is consistent with the topography images of wear shown in Figure 2. The large stick-slip peaks are too few to show up in the FT analysis, but from this analysis, we still notice that the distance between the smaller peaks remain larger at 75%RH than at 5%RH.

Thus, we conclude from the wear measurements that water adsorption has a significant effect on both wear of calcite and stick-slip between calcite and the tip. It appears that water makes the surface region somewhat softer and less brittle, resulting in larger stick-slip amplitude, stick-slip length, and reduced abrasive wear.

### Modified Calcite

**Figure 4:**
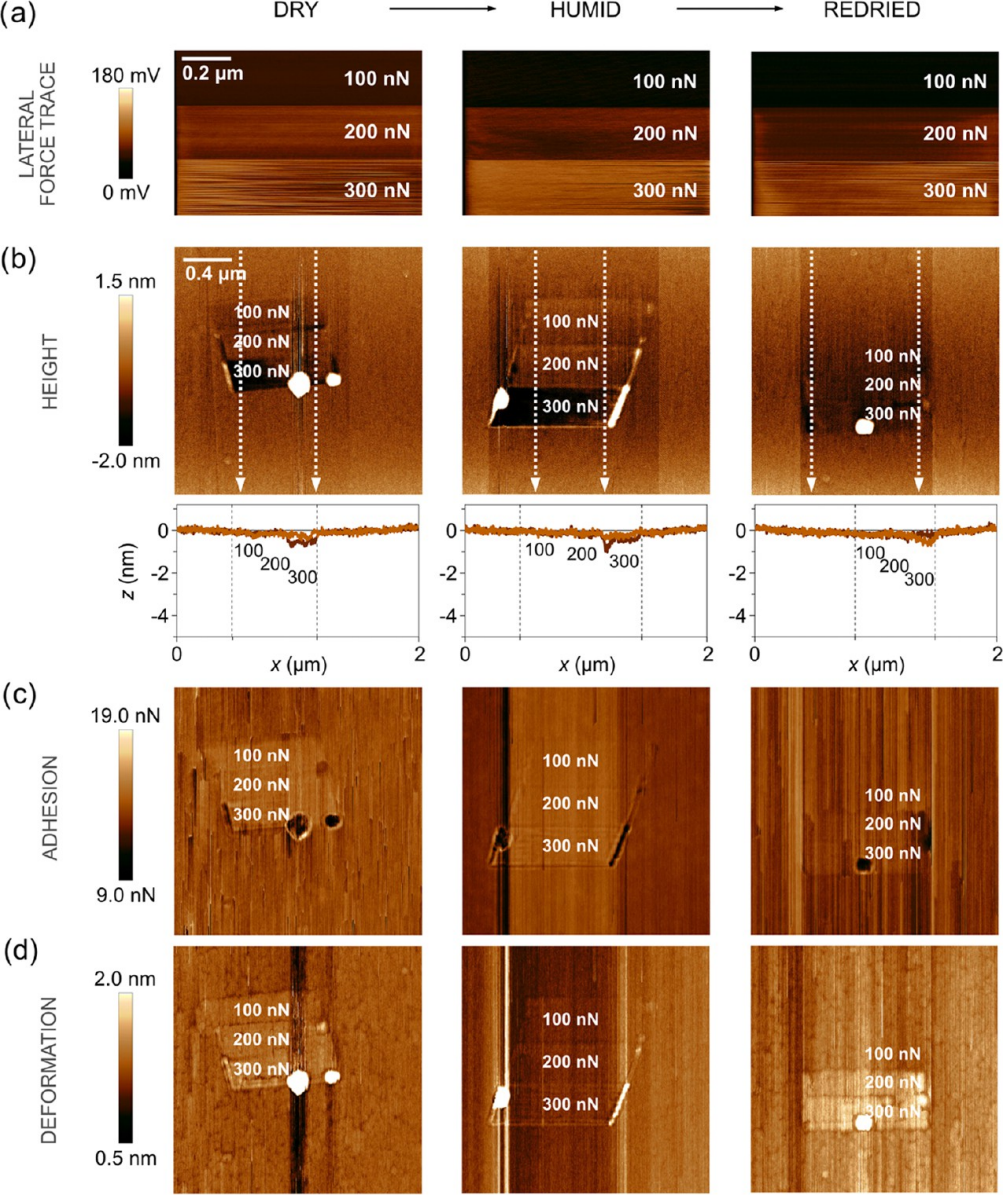
(a) Lateral force images (1 × 0.67 μm^2^), expressed in terms of lateral photodetector voltage, recorded during the wear measurements of stearic acid-modified calcite (4 h exposure to stearic acid vapor) under dry (<5%RH, left column), humid (≈75%RH, middle column), and redried (<5%RH, right column) conditions and increasing applied force. The sliding speed was 1 μm/s. (b) Topography images, followed by line scans across the worn area show the wear depth; below (c) adhesion images, and at the bottom row, (d) deformation images determined after the wear measurements. The scan size was 2 × 2 μm^2^, and the images contain 512 × 512 data points. Average roughness, adhesion, and deformation of worn and unworn areas are provided in Tables S1–S3 in the Supporting Information.

As judged from the homogeneous AFM image and a high water contact angle of ∼110°, calcite is completely covered by a stearic acid monolayer after 4 h exposure to its vapor at 105 °C. The presence of this layer affects the wear properties significantly. The wear scar on stearic acid-modified calcite (Figure 4) is more homogeneous than for bare calcite (Figure 2); however, at the highest load of 300 nN, wear particles consisting of stearic acid molecules are observed. Further, the wear depth does not exceed 1 nm even under a load of 300 nN, i.e., less than the thickness of the stearic acid monolayer. Such low wear and visibly homogeneous adhesion and deformation across the wear and at the nonworn area suggest that the stearic acid layer is sufficiently robust and well packed to withstand the forces used to disturb it. For stearic acid-modified calcite, the topographical wear scar is not much affected by the humidity, which also is different from that found for bare calcite. This is due to the hydrophobic nature of the stearic acid monolayer that prevents capillary condensation.

**Figure 5:**
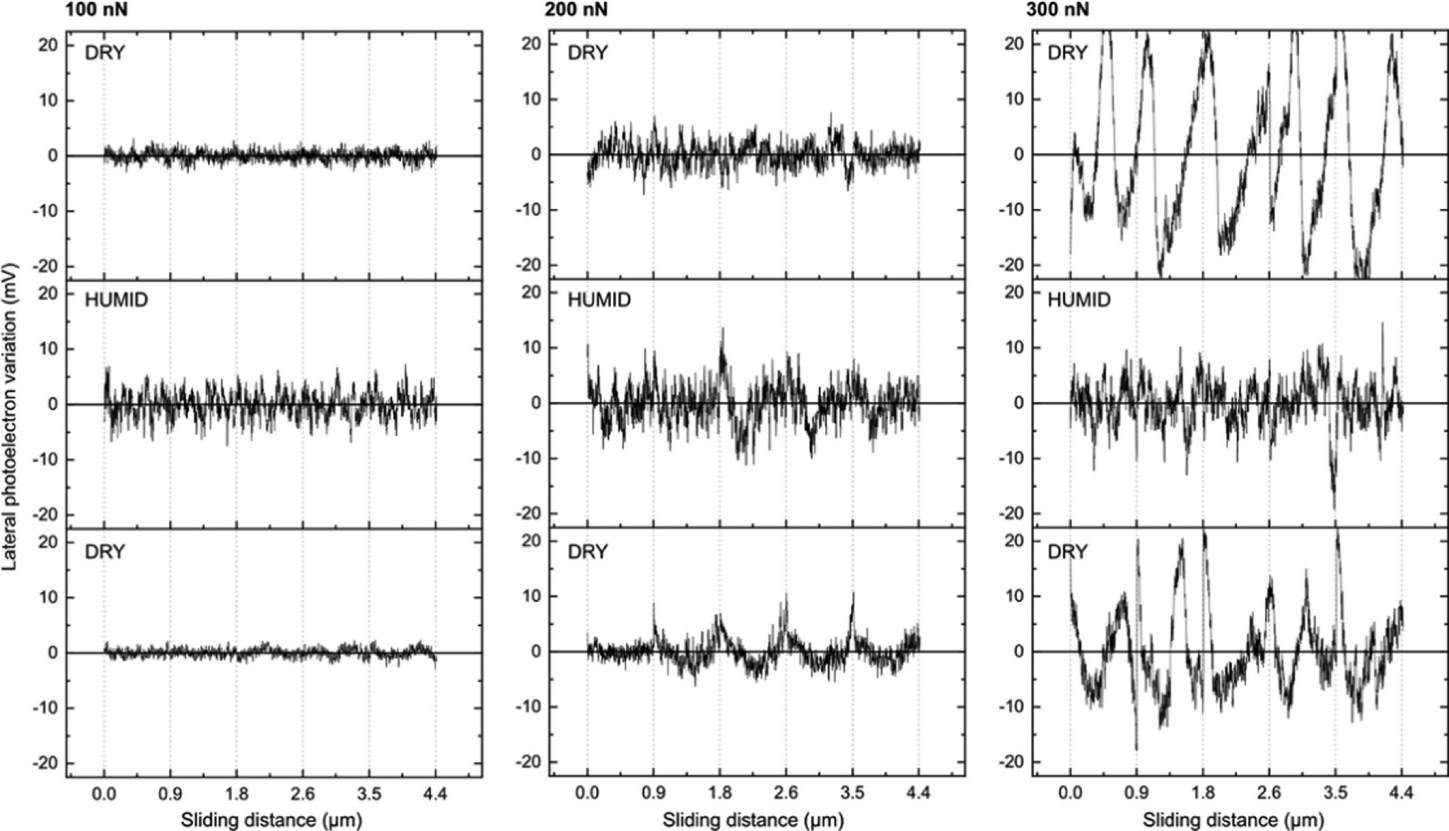
Lateral force variations during sliding reported for stearic acid-modified calcite (4 h vapor deposition) under dry (<5%RH, top row), humid (≈75%RH, middle row), and redried (<5%RH, bottom row) condition and rising applied force. The first column contains data recorded at a load of 100 nN, middle column data at 200 nN, and third column data at 300 nN. The sliding speed was 1 μm/s in all cases. One-dimensional Fourier transforms are shown in Figure S1 in the Supporting Information.

The lateral photodetector voltage variations and Fourier transforms of these are shown in Figures 5 and S1 in the Supporting Information. In these images, we note clear differences depending on the humidity, which is somewhat surprising since the wear scar is similar at <5 and 75%RH. At a low force, 100 nN, the stick-slip amplitude is clearly larger in humid air than in dry air. Following the interpretation given for bare calcite in Figure 3, the higher stick-slip amplitude at 75%RH suggests a softer stearic acid layer at a higher humidity. This effect is likely due to some water being present at the stearic acid–calcite interface that reduces the binding strength of the carboxylic acid.

At a high force, 300 nN, where some abrasive wear of the stearic acid layer occurs, larger stick-slip amplitudes are observed in dry air than in humid air. This finding can be rationalized as it is the stearic acid layer and not the calcite that wears. The larger stick-slip amplitude in dry state is thus due to the higher binding strength under dry conditions that requires a larger force for lateral displacement of the adsorbed molecules. In addition, the limited remaining wear scar suggests that once the tip has locally displaced some adsorbed stearic acid molecules, the layer partly reforms and heals the wear scar. Thus, it can be proposed that the stearic acid layer has a certain self-healing ability both under dry and humid conditions. More importantly, the presence of a stearic acid layer on the calcite surface makes the calcite surface itself less vulnerable to wear, and in our experiments, we find no evidence for wear of the calcite substrate when coated by a stearic acid monolayer.

From the Fourier analysis (Figure S1), we notice two peaks due to electronic noise at 0.05 and 0.1 nm^–1^, and the stick-slip lengths move toward smaller values of 1/*d* (larger distance) with increasing load. This is similar to that observed for bare calcite, but unlike what has been previously observed for polymer layers that deform plastically,^43−47^ there is no clear characteristic stick-slip length. Interestingly, two characteristic stick-slip lengths have been reported for adsorbed catechol-containing polymers, whereas adsorbed polyelectrolytes without catechol groups displayed one single characteristic stick-slip length.^28^

### Patchy Stearic Acid Layer

**Figure 6:**
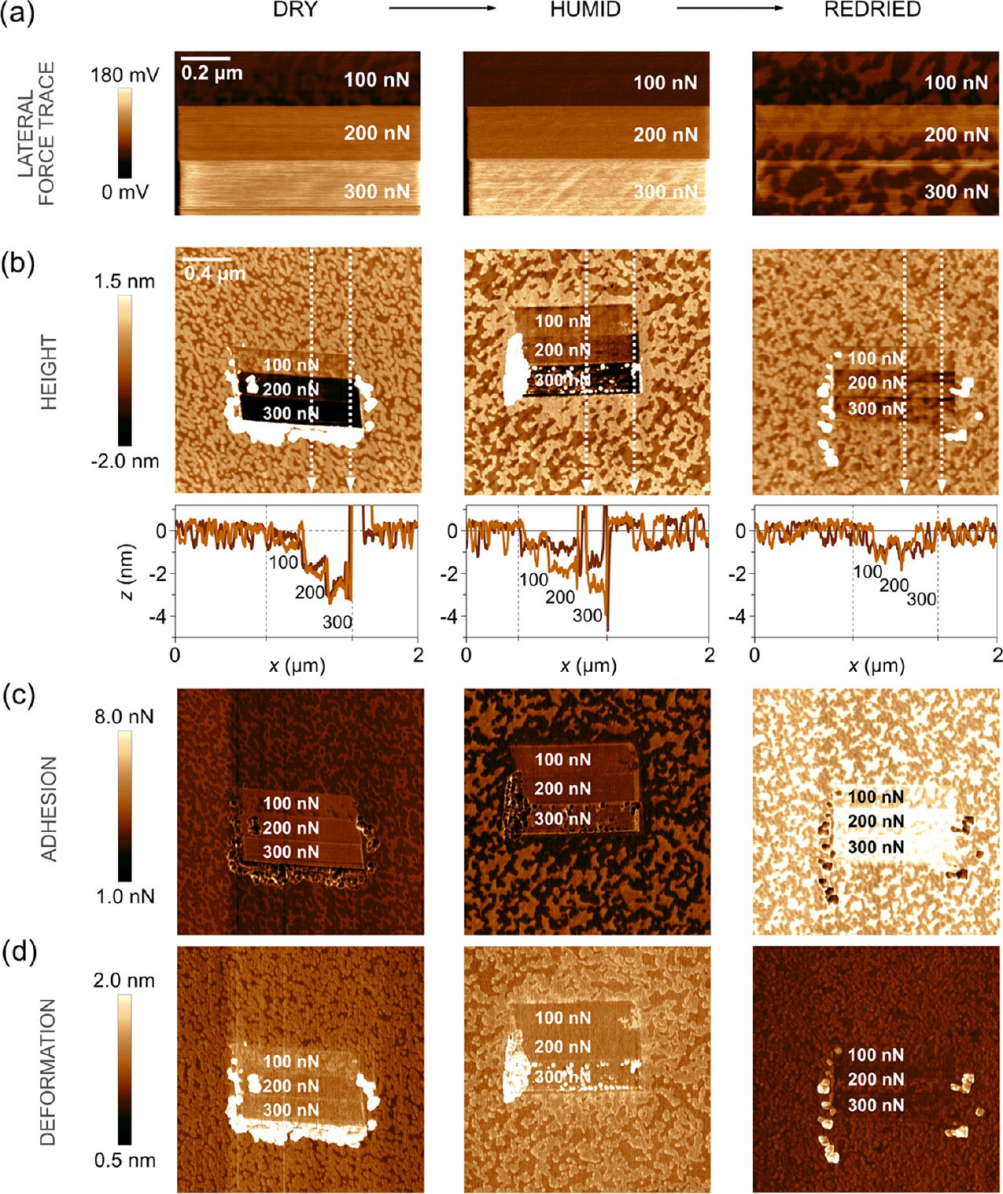
(a) Lateral force images (1 × 0.67 μm^2^), expressed in terms of lateral photodetector voltage, recorded during the wear measurements of calcite modified by exposure to stearic acid vapor for 10 min under dry (<5%RH, left column), humid (≈75%RH, middle column), and redried (<5%RH, right column) conditions and increasing applied force. The sliding speed was 1 μm/s. (b) Topography images, followed by line scans across the worn area that show the wear depth; below (c) adhesion images, and at the bottom row, (d) deformation images determined after the wear measurements. The scan size was 2 × 2 μm^2^, and the images contain 512 × 512 data points. Average roughness, adhesion, and deformation of worn and unworn areas are provided in Tables S1–S3 in the Supporting Information.

In this section, we consider the situation with an incomplete and patchy stearic acid layer that is formed during short time exposure (10 min) to stearic acid vapor at 105 °C, where the initial water contact angle was found to be ∼70°. The stearic acid patches can be distinguished by lower adhesion and higher deformation than the unmodified calcite, as illustrated in Figure 6. By comparing the deformation data reported in Figures 4 and 6, we note that the stearic acid layer is more deformable at the lower packing density. The stearic acid patches are also more deformable at 75%RH than at <5%RH. This is assigned to easier lateral motion along the surface at a lower packing density and higher humidity. The effect of wear is similar to that found for freshly cleaved and uncoated calcite with wear depth up to 4 nm at 300 nN. In the dry state (<5%RH), stearic acid patches withstand a load of 100 nN but are removed at 200 nN, whereas at 75%RH, the stearic acid patches are removed already at 100 nN. This clearly demonstrates that water vapor reduces the binding strength of stearic acid to calcite, as also proposed in the previous section.

At a low humidity, the wear scar is relatively smooth at forces of 200 and 300 nN. In contrast, an inhomogeneous wear scar is found at high forces at 75%RH, which was also observed for unmodified calcite.

**Figure 7:**
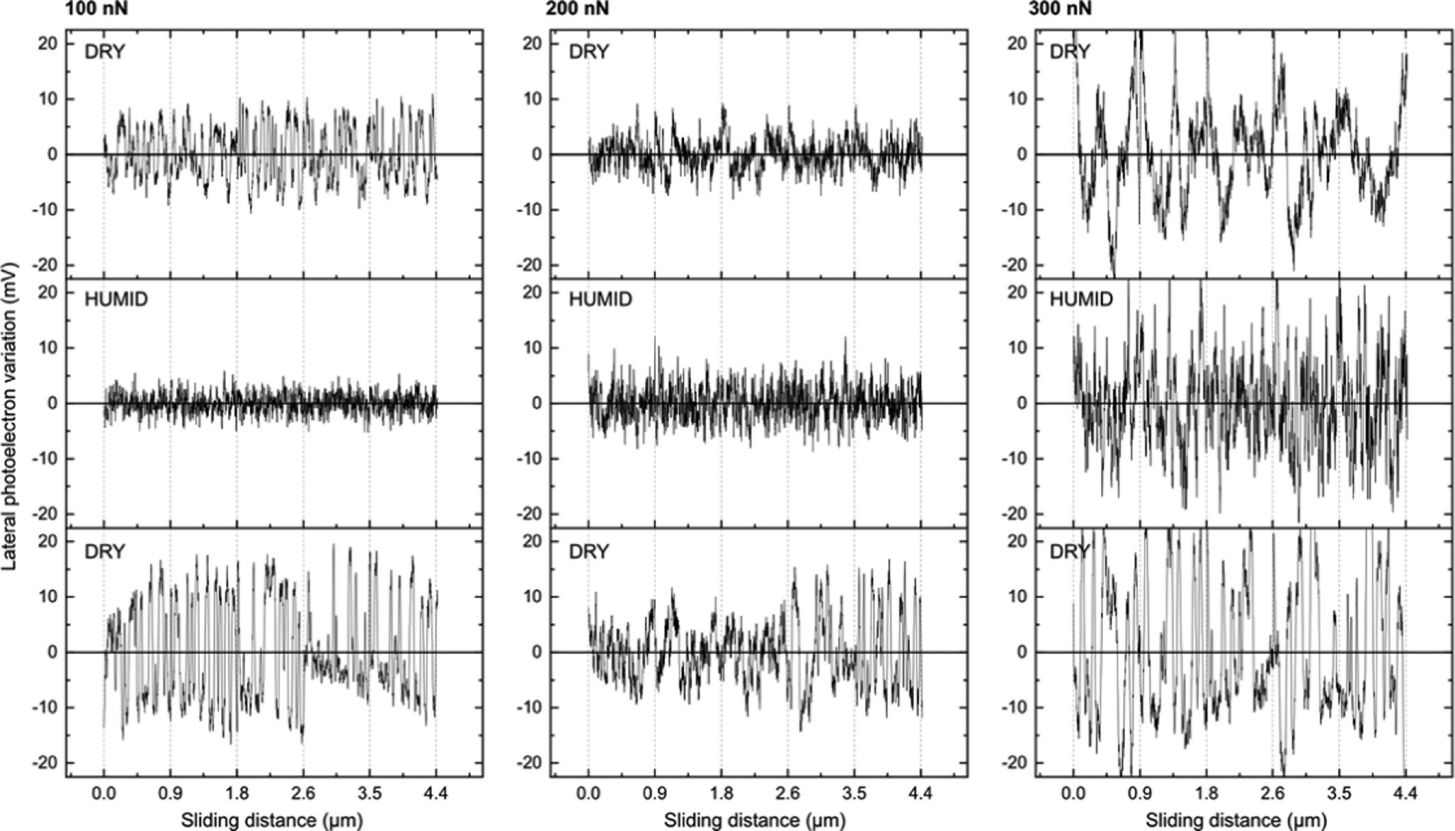
Lateral force variations during sliding reported for calcite carrying a patchy stearic acid layer under dry (<5%RH, top row), humid (≈75%RH, middle row), and redried (<5%RH, bottom row) conditions and increasing applied force. The first column contains data recorded at 100 nN, middle column data at 200 nN, and third column data at 300 nN. The sliding speed was 1 μm/s in all cases. One-dimensional Fourier transforms are shown in Figure S2 in the Supporting Information.

The effect of the patchy layer structure on the lateral force variation can be observed in Figure 7. The main difference, compared to bare calcite and CaCO_3_ modified with a complete layer of stearic acid, is the larger stick-slip amplitude at a low force (100 nN) under dry conditions. It is under this condition that abrasive wear is very limited as seen in the topography image and, thus, here the stick-slip amplitude is related to the inhomogeneous surface structure. We note that the stick-slip amplitude is lower under humid conditions where the binding strength of stearic acid to calcite is reduced. At higher forces, abrasive wear of the calcite substrate becomes important, and the stick-slip pattern becomes similar to that observed for unmodified calcite.

### Friction Coefficients

**Figure 8:**
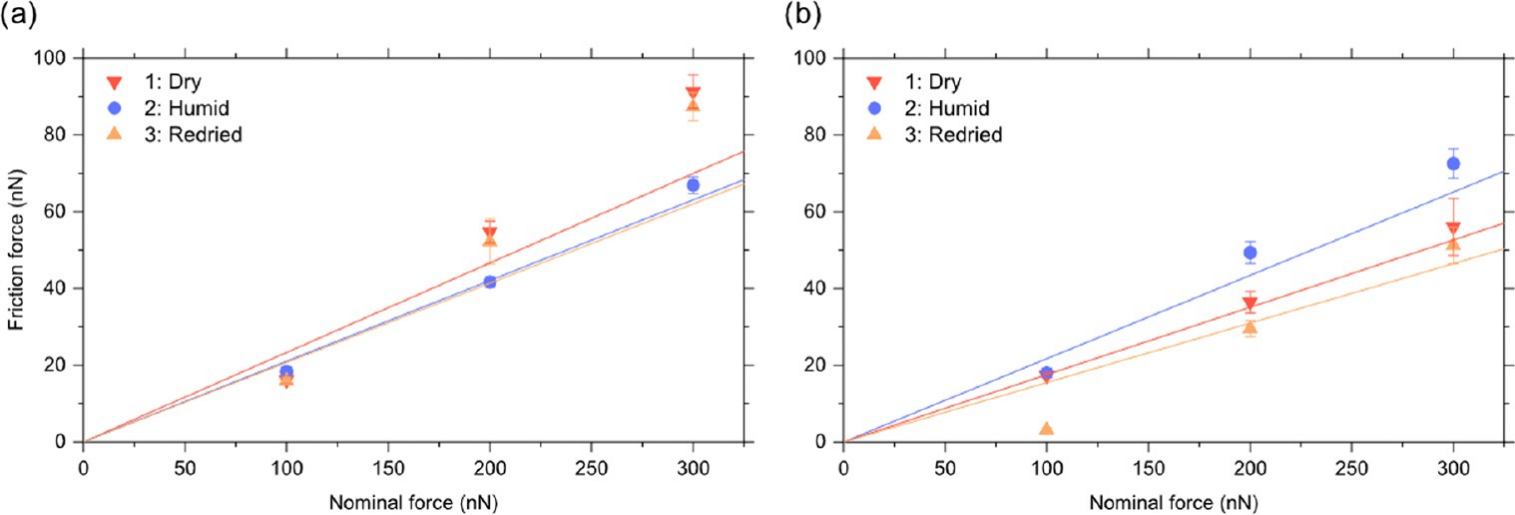
Friction force as a function of load under dry (<5%RH), humid (≈75%RH), and again dry (<5%RH) conditions for (a) freshly cleaved and unmodified calcite and (b) calcite modified by exposure to stearic acid vapor for 4 h at 105 °C.

In many, but not all, cases, the friction force, *F*_f_, depends linearly on the applied load, *F*_n_, according to Amontons’ rule
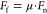
3where the proportionality constant μ is the coefficient of friction. When this rule applies, one expects to see a straight line going through the origin when the friction force is plotted against the load. The data shown in Figure 8 agrees reasonably, but far from perfectly, with predictions based on Amontons’ rule. This disagreement is hardly surprising considering that our measurements contain data points in load regions with no significant wear as well as with substantial abrasive wear. Thus, the main energy dissipative mechanism is different at low loads where the tip slides on top of the surface and causes surface deformation and at high loads where the action of the tip disintegrates the surface. The friction coefficients evaluated using Amontons’ rule is provided in Table 1. An intriguing observation is the low friction force repeatedly found at 100 nN for the redried stearic acid-modified calcite. In a vibrational sum frequency study that will be reported elsewhere, we found that the vapor-deposited layer of stearic acid consists of a mixture of the acid form and the carboxylate form. It is plausible that the presence of water facilitates the conversion of the acid form to the carboxylate form, which results in additional strong calcium carboxylate bonds that increase the wear resistance of the adsorbed layer and lowers the friction force. However, this hypothesis remains to be tested. In the fitting to eq 3, the low friction point at 100 nN for the redried case in Figure 2 was not considered.

**Table 1:** Friction Coefficients, μ, Evaluated Based on Amontons’ Rule

	<5%RH	≈75%RH	<5%RH (redried)
calcite	0.23	0.21	0.21
calcite modified by stearic acid	0.18	0.22	0.16

For bare calcite at a load of 100 nN, where the abrasive wear is low, the relative humidity hardly affects the friction force even though the stick-slip amplitude was affected by the humidity. Clearly, the about 1.5 nm thin^39^ adsorbed water layer at 75%RH has no noticeable lubricating effect. However, at higher loads, the friction force is lower at 75%RH than under the dry condition. The reason for this is the more pronounced abrasive wear observed under dry conditions (Figure 2), which, in turn, is related to the higher brittleness of dry calcite compared to hydrated forms of calcium carbonate that form on the surface at a high humidity.

The situation for calcite modified with a complete layer of stearic acid is opposite, and the friction force is higher at 75%RH than in <5%RH. The reason is that the main energy dissipative mechanism arises from displacing the stearic acid molecules on the surface, and more molecules are displaced during shearing in humid conditions due to the lower binding strength of stearic acid to calcite in the presence of water vapor. This also rationalizes why the stearic acid layer reduces the friction force in dry state but slightly increases it in humid state compared to the situation with unmodified calcite.

## Conclusions

We have elucidated the local wear characteristics of unmodified and stearic acid-modified calcite surfaces in dry and humid air. The results demonstrate that a stearic acid monolayer is capable of preventing wear of the calcite surface in both dry and humid air under the loads explored (up to 300 nN). The data also demonstrate the importance of packing density of the stearic acid layer and the air humidity.

Abrasive wear of unmodified calcite was found to be negligible at an applied force of 100 nN under both dry and humid conditions (≈75%RH). The exception is that recrystallized patches on the calcite surface were removed (Figure 2). During wear measurements, an irregular stick-slip sliding is observed, where both the stick-slip amplitude and stick-slip length are higher under the more humid condition. This suggests a softening of the calcite surface due to water adsorption. At higher applied forces (200–300 nN), abrasive wear is significant for unmodified calcite in both dry and humid states. The wear depth is as much as 4 nm. In the dry state, wear particles are pushed to the side of the worn area, whereas many wear particles remain in the worn area under humid conditions. This difference is suggested to be due to capillary forces between wear particles and the calcite surface. The friction force between the AFM tip and the unmodified calcite surface was not affected by the humidity. Thus, adsorbed water molecules under humid conditions have no significant lubricating effect.

A full coverage of stearic acid on the calcite surface was found to significantly reduce the wear, and the wear depth did not exceed 1 nm, i.e., less than the thickness of the monolayer, even at 300 nN load under dry and humid conditions. Clearly, the stearic acid modification is robust, and it protects the calcite surface from wear and the limited abrasive wear observed largely occurs within the adsorbed layer. Nevertheless, the sliding at high forces was characterized by large stick-slip amplitudes. The fact that the wear depth despite this remains small suggests that the stearic acid layer has a certain self-healing ability.

A patchy coverage of stearic acid did not have the same beneficial effect on the wear, but in this case, the wear properties were largely similar to those of bare calcite. However, the inhomogeneous layer structure remained after challenged by a load of 100 nN combined with shear under dry conditions, which gave rise to large stick-slip amplitudes. In contrast, the inhomogeneous stearic acid layer was worn away at the same load at 75%RH. This implies stronger binding of the stearic acid layer under dry conditions.

The friction force data show that the presence of a complete layer of stearic acid on the calcite surface slightly reduces the friction force at a low humidity, but it has no impact at 75%RH. An intriguing observation is that a stearic acid-modified surface that was exposed to 75%RH and then redried displayed a significantly lower friction force at 100 nN (prior to abrasive wear) than a similar surface not exposed to high humidity. We speculate that this is due to water-induced reactions where protonated stearic acid molecules are converted to carboxylate form that subsequently reacts to form strongly bound calcium carboxylates upon drying.

To summarize, a complete stearic acid layer provides improved wear resistance, while a patchy one does not provide any significant benefit. Thus, it is important to achieve full stearic acid coverage not only from wetting and surface energy considerations but also from wear perspectives.

## Supporting Information Available

One-dimensional Fourier transforms of the data reported for modified calcite for 10 min and 4 h with C_18_ acid; average roughness parameters (*R*_q_, *R*_a_, *R*_max_ [nm]) for height in wear marks compared to the initial surface; mean value (nN) and standard deviation, Stdev, (nN) for adhesion in wear marks compared to the initial surface; and mean value (nm) and standard deviation, Stdev, (nm) for deformation in wear marks compared to the initial surface (PDF)
