# Supplementary material for: Nanoscale Wear and Mechanical Properties of Calcite: Effects of Stearic Acid Modification and Water Vapor
Source: Langmuir. 2021 Aug 6;37(32):9826–37. doi: 10.1021/acs.langmuir.1c01390 (PMC8397405; doi:10.1021/acs.langmuir.1c01390)
Supplement: Supplementary file 1 — la1c01390_si_001.pdf [file la1c01390_si_001.pdf]

Nano-scale wear and mechanical properties of calcite: Effects of stearic acid modification and water vapor

Natalia A. Wojas [a,b,*], Illia Dobryden [b,c], Viveca Wallqvist [a], Agne Swerin [d], Mikael Järn [a], Joachim Schoelkopf [e], Patrick A.C. Gane [f], Per M. Claesson [a,b,*]

[a] RISE Research Institutes of Sweden, Bioeconomy and Health Division, Department Materials and Surface Design, Box 5607, SE - 114 86 Stockholm, Sweden
[b] KTH Royal Institute of Technology, School of Engineering Sciences in Chemistry, Biotechnology and Health, Department of Chemistry, Division of Surface and Corrosion Science, Drottning Kristinas väg 51, SE - 100 44 Stockholm, Sweden
[c] Department of Engineering Sciences and Mathematics, Division of Materials Science, Luleå University of Technology, SE – 971 87 Luleå, Sweden
[d] Karlstad University, Faculty of Health, Science and Technology, Department of Engineering and Chemical Sciences: Chemical Engineering, SE - 651 88 Karlstad, Sweden
[e] Omya International AG, Baslerstrasse 42, CH - 4665 Oftringen, Switzerland
[f] Aalto University, School of Chemical Engineering, Department of Bioproducts and Biosystems, P.O. Box 16300, FI - 00076 Aalto, Finland

* Corresponding authors email: natalia.anna.wojas@ri.se and percl@kth.se

SUPPORTING INFORMATION

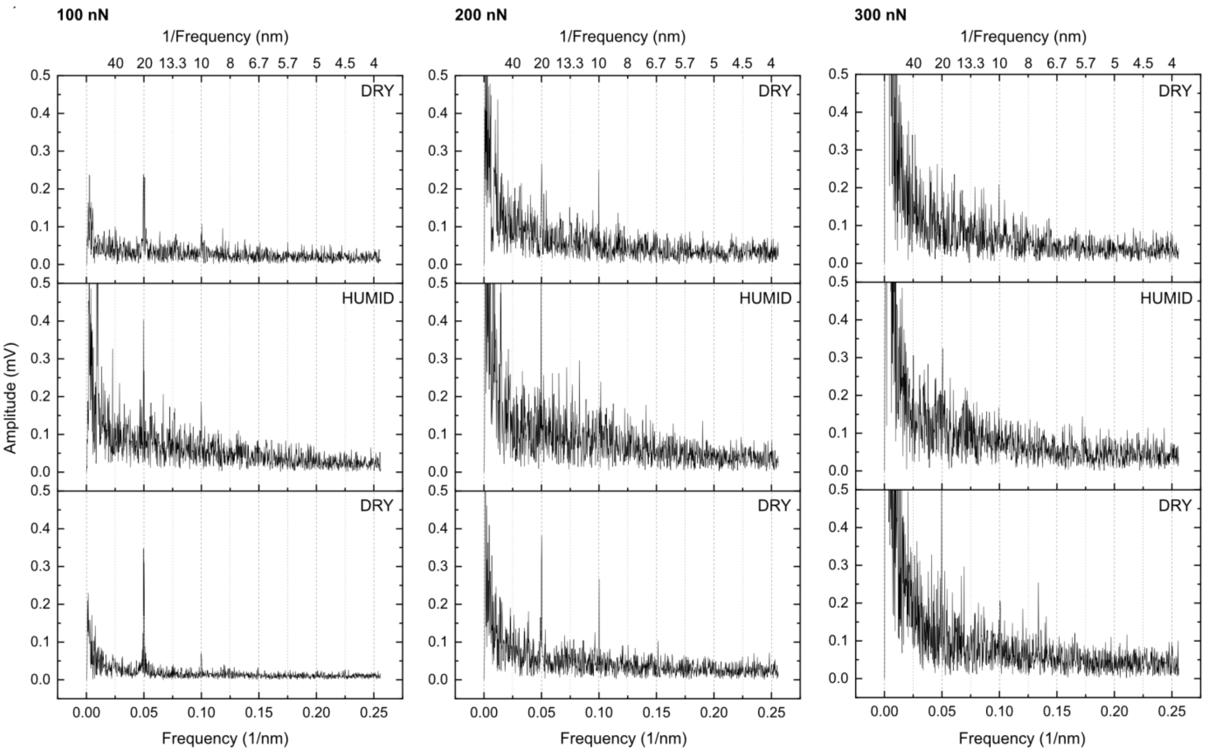

Figure S1. One dimensional Fourier transforms of the data reported for stearic acid modified calcite (4 h vapor deposition) at dry (< 5 %RH, top row), humid (≈ 75 %RH, middle row) and redried (< 5 %RH, bottom row) condition and rising applied force. First column contain data recorded at a load of 100 nN, middle column data at 200 nN, and third row data at 300 nN. The sliding speed was 1 μm/s in all cases.

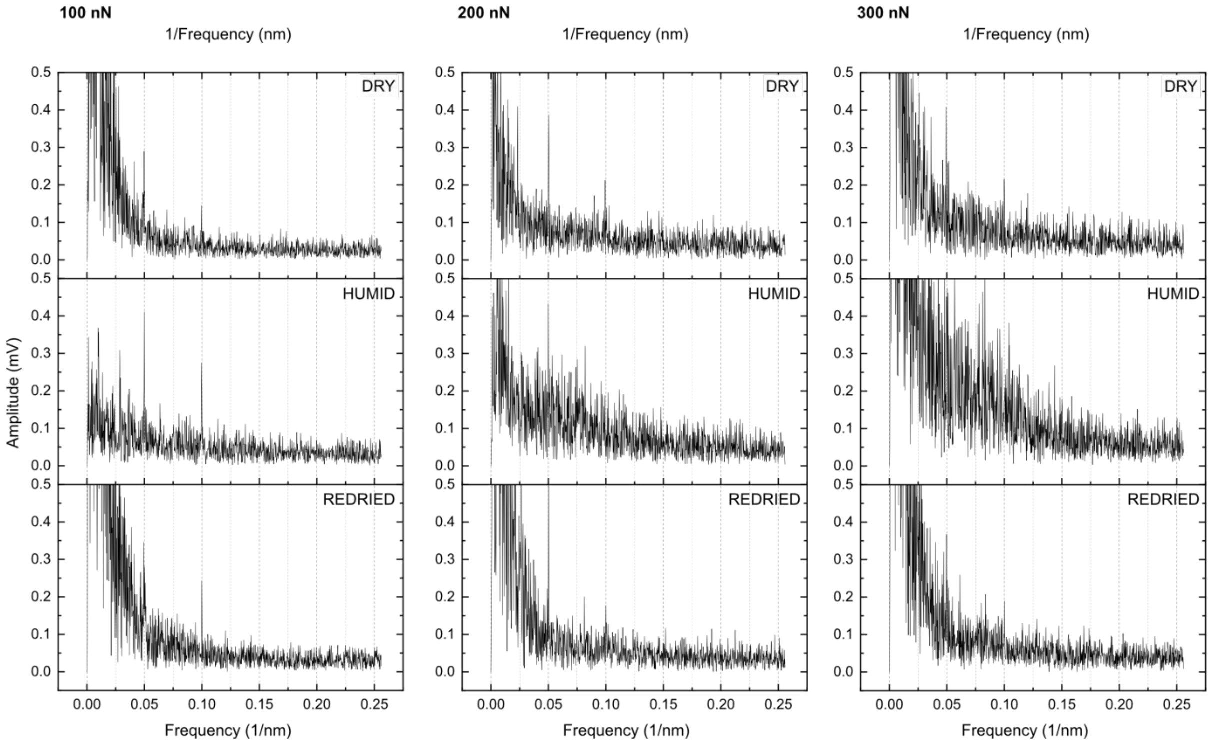

Figure S2. One dimensional Fourier transforms of the data reported for modified calcite for 10 min with C_{18} at dry (< 5 %RH, top row), humid (≈ 75 %RH, middle row) and redried (< 5 %RH, bottom row) condition and rising applied force. First column contain data recorded at 100 nN, middle column data at 200 nN, and third row data at 300 nN. The sliding speed was 1 µm/s in all cases.

Table S1.

Average roughness parameters (R_q, R_a, R_{max} [nm]) for height in wear marks compared to the initial surface. Data are shown for bare calcite and calcite modified with stearic acid at full coverage (4 h vapor deposition) and with a patchy coverage (10 min vapor deposition).

Bare calcite				Modified calcite (4 h C_{18})				Modified calcite (10 min C_{18})			
**Surface type**	R_q	R_a	R_{max}	**Surface type**	R_q	R_a	R_{max}	**Surface type**	R_q	R_a	R_{max}
**DRY**				**DRY**				**DRY**			
initial	0.10	0.08	0.82	initial	0.10	0.08	0.90	initial	0.37	0.32	1.98
100 nN	0.09	0.07	0.87	100 nN	0.08	0.07	0.66	100 nN	0.26	0.21	1.63
200 nN	0.40	0.31	2.52	200 nN	0.08	0.07	0.65	200 nN	0.16	0.13	1.86
300 nN	0.51	0.41	2.98	300 nN	0.11	0.08	0.81	300 nN	0.40	0.29	3.65
**WET**				**WET**				**WET**			
initial	0.09	0.07	0.83	initial	0.11	0.09	0.85	initial	0.54	0.49	2.77
100 nN	0.15	0.12	0.96	100 nN	0.09	0.07	0.73	100 nN	0.25	0.19	2.27
200 nN	0.40	0.31	3.39	200 nN	0.09	0.07	0.76	200 nN	0.24	0.19	2.81
300 nN	1.11	0.84	6.62	300 nN	0.13	0.10	1.43	300 nN	1.63	0.99	14.70
**REDRIED**				**REDRIED**				**REDRIED**			
initial	0.10	0.08	0.88	initial	0.09	0.07	0.63	initial	0.35	0.30	2.06
100 nN	0.09	0.07	0.75	100 nN	0.09	0.07	0.61	100 nN	0.33	0.28	1.85
200 nN	0.46	0.38	2.24	200 nN	0.09	0.07	0.71	200 nN	0.27	0.22	1.99
300 nN	0.50	0.38	2.81	300 nN	0.11	0.08	0.93	300 nN	0.30	0.24	2.19

Table S2.

Mean value (nN) and standard deviation, Stdev, (nN) for adhesion in wear marks compared to the initial surface. Data are shown for bare calcite and calcite modified with stearic acid at full coverage (4 h vapor deposition) and with a patchy coverage (10 min vapor deposition).

Bare calcite			Modified calcite (4 h C_{18})			Modified calcite (10 min C_{18})		
**Surface type**	**Mean**	**Stdev**	**Surface type**	**Mean**	**Stdev**	**Surface type**	**Mean**	**Stdev**
**DRY**			**DRY**			**DRY**		
initial	3.6	0.3	Initial	13.6	0.7	initial	3.1	0.3
100 nN	3.8	0.2	100 nN	14.0	0.7	100 nN	3.6	0.2
200 nN	3.8	0.2	200 nN	14.0	0.6	200 nN	3.7	0.1
300 nN	3.7	0.2	300 nN	14.1	0.5	300 nN	3.7	0.1
**WET**			**WET**			**WET**		
initial	3.4	0.2	Initial	13.8	0.3	initial	3.2	0.8
100 nN	3.2	0.2	100 nN	14.2	0.2	100 nN	3.5	0.2
200 nN	3.1	0.2	200 nN	14.3	0.3	200 nN	3.3	0.2
300 nN	2.9	0.4	300 nN	14.4	0.3	300 nN	3.4	0.7
**REDRIED**			**REDRIED**			**REDRIED**		
initial	3.1	0.3	Initial	13.4	0.6	initial	7.5	0.9
100 nN	3.3	0.2	100 nN	13.5	0.7	100 nN	8.1	0.8
200 nN	3.3	0.2	200 nN	13.6	0.6	200 nN	8.7	0.5
300 nN	3.4	0.2	300 nN	13.6	0.7	300 nN	8.8	0.3

Table S3.

Mean value (nm) and standard deviation, Stdev, (nm) for deformation in wear marks compared to the initial surface. Data are shown for bare calcite and calcite modified with stearic acid at full coverage (4 h vapor deposition) and with a patchy coverage (10 min vapor deposition).

Bare calcite			Modified calcite (4 h C_{18})			Modified calcite (10 min C_{18})		
**Surface type**	**Mean**	**Stdev**	**Surface type**	**Mean**	**Stdev**	**Surface type**	**Mean**	**Stdev**
**DRY**			**DRY**			**DRY**		
initial	1.22	0.09	Initial	1.39	0.14	initial	1.21	0.11
100 nN	1.43	0.11	100 nN	1.47	0.13	100 nN	1.46	0.10
200 nN	1.50	0.10	200 nN	1.49	0.13	200 nN	1.36	0.09
300 nN	1.46	0.11	300 nN	1.51	0.16	300 nN	1.40	0.10
**WET**			**WET**			**WET**		
initial	1.37	0.09	initial	0.95	0.06	initial	1.46	0.13
100 nN	1.40	0.10	100 nN	0.99	0.05	100 nN	1.40	0.08
200 nN	1.41	0.10	200 nN	1.01	0.05	200 nN	1.39	0.08
300 nN	1.53	0.12	300 nN	1.02	0.06	300 nN	1.54	0.28
**REDRIED**			**REDRIED**			**REDRIED**		
initial	1.37	0.10	initial	1.51	0.10	initial	0.22	0.06
100 nN	1.48	0.09	100 nN	1.57	0.09	100 nN	0.21	0.05
200 nN	1.53	0.08	200 nN	1.70	0.09	200 nN	0.21	0.06
300 nN	1.54	0.08	300 nN	1.78	0.10	300 nN	0.19	0.05
